# Clinical and pathological factors and outcome of central nervous system metastasis in breast cancer

**DOI:** 10.3389/fonc.2023.1247402

**Published:** 2023-09-19

**Authors:** Dimitri Dettwiler, Elena-Diana Chiru, Eveline Daetwyler, Vérène Dougoud-Chauvin, Markus W. Gross, Christian Kurzeder, Alfred Zippelius, Andreas Schötzau, Marcus Vetter

**Affiliations:** ^1^ Department of Medical Oncology, University Hospital Basel, Basel, Switzerland; ^2^ Cancer Center, Medical University Clinics, Kantonsspital Baselland, Liestal, Switzerland; ^3^ Department of Medical Oncology, Kantonsspital HFR Freiburg, Fribourg, Switzerland; ^4^ Department of Radiooncology, University Hospital Basel, Basel, Switzerland; ^5^ Breast Center, University Hospital Basel, Basel, Switzerland; ^6^ Department of Gynecologic Oncology, University Hospital Basel, Basel, Switzerland; ^7^ Cancer Center Baselland, Medical University Clinic Baselland, Liestal, Switzerland; ^8^ Medical Faculty University Basel, Basel, Switzerland

**Keywords:** metastatic breast cancer, brain metastasis, meningeal carcinomatosis, older patients with breast cancer, long-term survivors with breast cancer and brain metastases

## Abstract

**Background:**

In Switzerland, approximately 6000 new breast cancer cases and 1300 deaths are reported annually. Brain metastasis from breast cancer (BMBC) has a major effect on prognosis. This study aimed to identify prognostic factors for overall survival (OS) in a cohort of Swiss patients with BMBC. This study evaluated the prognosis on older BMBC, which has not been completely addressed in the literature.

**Methods:**

We performed a retrospective chart review analysis with the primary endpoint of OS after a diagnosis of BMBC. The study population was divided into 2 groups based on an OS cut-off value of 12 months after diagnosis. Univariate and multivariate analyses of several risk factors, including age, were performed. To evaluate differences in OS according to age, we performed a secondary analysis to examine the prognostic value of clinical symptoms, metastatic pattern, and lymph node involvement in an older (≥65 years) vs. younger (<65 years) cohort.

**Results:**

From 1989 to 2019, 55 patients were identified as having BMBC, among whom 47 patients were confirmed to be dead. The median patient age was 58 years (range 25–83 years). Comorbidities were present in 45 (81.8%) patients. The median survival in the OS <12 and OS ≥12 months groups was 4.3 and 30.7 months, respectively (*p*<0.001). Multivariate analysis revealed no significant differences in terms of comorbidities, medication use, M-stage, and symptomatology between the 2 groups. Additionally, there was no significant difference in OS in the 2 subgroups of patients aged <65 and ≥65 years.

**Discussion:**

We concluded that age should not be a decisive factor in therapy planning for advanced breast cancer patients with BMBC.

## 1 Introduction

Breast cancer (BC) is a global healthcare burden and was the most diagnosed cancer in women in 2020, accounting for 25% of newly diagnosed cancers worldwide. An estimated 2.2 million new BC cases occurred worldwide in the same year (1).

In Switzerland, the annual incidence of newly diagnosed BC in women is approximately 6000 cases, with an annual death rate of 1300 cases (1). Around 3–6% of all patients with newly diagnosed primary BC in high income countries have synchronous metastatic disease (Stage IV) (2, 3).

Lymph nodes, bones, liver, lungs, and the central nervous system (CNS) are among the most frequent locations of metastasis (4). Earlier stage disease shows varying risks of recurrence or metastasis depending on several factors such as molecular subtype, tumor size, histologic grade, and proliferation rate (Ki-67) (5, 6). In patients with early stage, hormone receptor (HR)-positive BC, the recurrence rate at 10 years is around 20% (7), and this risk remains high for decades (8). In contrast, the highest risk of recurrence of triple-negative BC (TNBC) is within the first 3 years after surgery. Similarly, patients with HER2-positive BC have the highest risk of recurrence within the first 5 years after diagnosis (8, 9).

Metastasis to the CNS is associated with poor prognosis, regardless of the cancer type (10, 11). The frequency of brain metastasis from BC (BMBC) ranges from 5% to 50% and has a major effect on patient survival (12–17). The median overall survival (OS) of patients following the diagnosis of cerebral metastasis ranges from 3.5 to 9 months, with a 1-year-survival rate of approximately 20% (10, 18, 19).

The risk and prognosis of CNS metastasis differ depending on the BC subtype. Approximately 7–15% of all patients with HR-positive BC will develop CNS metastasis during the course of their disease (6, 8). Higher rates of brain metastasis are observed in patients who are positive for HER2 and who have TNBC. According to the literature, 20–50% of all patients with HER2-positive BC (at primary advanced stage) and 25–46% of patients with TNBC will develop brain metastasis (8, 10, 14–17, 19, 20).

Voduk et al. showed that the risk of relapse in BC can be dependent on molecular subtype (20). Likewise, several risk factors for the development of BMBC have been identified, such as diagnosis at young age, positive lymph node status, and liver metastasis (21). Furthermore, positive HER2 status, lack of estrogen receptor expression (ER-), ductal tumor histology, small tumor size (T1/2), and M0 metastatic status were identified as risk factors for the development of cerebral metastasis by Heitz et al. (19). There are also well-established prognostic factors for patients diagnosed with brain metastasis, such as molecular subtype (better prognosis for positive HER2 and/or ER status), early detection of brain metastasis (within 6 months after diagnosis of metastatic disease), and asymptomatic disease, all of which are associated with longer OS (22).

Treatment of BMBC in older patients is challenging; therefore, it is likely that this group of patients has worse prognosis. Although most patients with BMBC are diagnosed at around 48–55 years of age (12, 23–26), no differences have been reported between the molecular subtypes with respect to age at the diagnosis of BMBC (26, 27). There have been conflicting reports regarding age as a possible independent prognostic risk factor in patients with BMBC. In the literature review by Rostami et al., there was no association between age and prognosis (28), while in a retrospective analysis by Bir et al., patients aged ≤65 years had better OS (12). Therefore, this issue remains controversial.

Hence, in this retrospective, single-center study, we focused on clinical outcomes of patients with BMBC. In Switzerland, all patients with brain metastasis are treated at dedicated cancer centers where all treatment modalities are available. The primary aim was to analyze outcomes (OS and progression-free survival [PFS]) among patients with BMBC and assess the prognostic value of specific factors described in the literature and seen in our population. The secondary aim was to determine differences in treatment allocation among younger and older patients with BMBC. The tertiary aim was to investigate risk factors associated with poorer (OS <12 months) and better prognosis (OS >12 months).

## 2 Methods

This retrospective chart review was conducted at the University Hospital of Basel, Switzerland between 1989 and 2019. Only patients with histologically confirmed BC and radiologically confirmed BMBC, with BC being the primary cancer, were included. The histological findings of brain metastases were reviewed where available. Patients without a clear histological diagnosis of BC or unclear cerebral metastases (for example, with additional malignancies as possible primaries) were excluded. Clinical and pathological data were collected using an electronic hospital information system. Patient data were pseudonymized and transferred to an MS Excel spreadsheet.

### 2.1 Endpoints and analysis

The primary endpoint of the study was the assessment of OS after the diagnosis of BMBC. Subsequent multivariate analysis to identify risk factors (e.g., age, molecular subtype, primary vs. secondary metastatic disease, prior cancer therapy, therapy for metastatic disease) in patients with poor prognosis (OS <12 months after diagnosis of CNS metastasis) and long-term survivors (OS ≥12 months after diagnosis of CNS metastasis) was performed. Further multivariate analysis was performed according to comorbidities, which were divided into lung and heart disease, diabetes mellitus (type 1 or 2), chronic infections (e.g., HIV), arterial hypertension, and peripheral artery disease, among others.

In a secondary analysis, we assessed outcome according to age (age <65 vs. ≥65 years), symptoms (asymptomatic vs. symptomatic), metastatic pattern, and lymph node involvement.

### 2.2 BC subtypes

BC subtypes were subdivided according to the St. Gallen International Breast Cancer Conference and ASCO CAP-Guidelines 13 into luminal A-like (ER+ and/or PR+ and HER2− and low proliferation factor), luminal B-like (ER+ and/or PR+ and/or HER2- and high proliferation factor), triple-negative BC (ER−/PR−/HER2−), and HER2-enriched (ER− and PR−/HER2+) (29).

### 2.3 Statistical analyses

Patient characteristics are summarized using descriptive statistics. Statistical significance was set at *p* values <0.05. The Kaplan–Meier method was used to calculate OS, and a log-rank test was used to compare OS between the groups. Markers that appeared to be significant using the log-rank test were included in a Cox regression model to predict OS, together with other known prognostic factors (e.g., age, stage, grading, and Ki-67). Patient age indicated was at the time of initial diagnosis of BC. For each patient, OS was measured from the date of diagnosis of CNS metastasis until the date of death. Patients without recorded death dates were censored on the date of the last available follow-up.

### 2.4 Ethical approval

The local ethics board (EKNZ, Number 2020-03023) approved this study. Patient`s consent was waived.

## 3 Results

### 3.1 Baseline characteristics

From 1989 to 2019, 55 patients with BMBC treated at the University Hospital Basel, Switzerland were included in this study. Approximately 200 patients diagnosed with BC were treated at our hospital. The total number of patients with brain metastasis possibly differed because of other treatments in hospitals outside Basel. At the time of analysis, 3 patients were still alive, while the time of death for 5 patients was unclear due to lack of data. The median age of the patients at the time of diagnosis was 58 years (range 25–83 years).

### 3.2 Comorbidities and medication

Comorbidities were present in 45 (82%) patients, and 7 (18%) patients had no previous history of comorbidities (**Table 1**). Unassigned secondary diseases (marked as other) were present in 63% of patients at time of primary diagnosis. In addition, 21 (38%) patients had arterial hypertension and 6 (11%) patients had diabetes mellitus at diagnosis.

**Table 1:** Baseline characteristics of patients with brain metastasis from breast cancer.

	n (%)
Age	n = 55
<65 years	41 (74.5%)
≥65 years	14 (25.5%)
Comorbidities	
Yes	45 (82%)
No	10 (18%)
Lung diseases	7 (13%)
Heart diseases	6 (11%)
Diabetes mellitus	6 (11%)
Arterial hypertension	21 (32%)
Peripheral artery disease	2 (4%)
Medications	n = 55
Yes	9 (16%)
No	45 (82%)
BC subtype	n = 55
Luminal A	1 (2%)
Luminal B	20 (36%)
Triple negative	11 (20%)
HER2/neu +	8 (15%)
Unclear	15 (27%)
Lymph node involvement at diagnosis of BC	n = 38
No lymph node involvement	17 (45%)
Lymph node involvement	21 (55%)
M-Stage at diagnosis of BC	n = 55
M0	33 (60%)
M1	21 (38%)
Pathological grade	n=55
G1 (low grade)	2 (3.6%)
G2	12 (21.8%)
G3 (high grade)	33 (60%)
No grading data	8 (14.5%)
Anatomical site of BM	n = 55
Cerebrum	19 (34.5%)
Cerebellum	1 (1.8%)
Meningeal carcinomatosis	5 (9%)
Spine	1 (1.8%)
More than one anatomical site	27 (49%)
Unclear	2 (3.6%)
Symptomatic brain metastases	n=55
Yes	42 (76%)
No	6 (11%)
Unclear	7 (13%)
Signs of midline shift at diagnosis of BMBC	
Yes	7 (13%)
No	48 (87%)
Signs of brain pressure at diagnosis of BMBC	
Yes	6 (11%)
No	49 (89%)

**Table 1** shows the clinical variables of all patients in the breast cancer and brain metastasis cohort. BC, breast cancer; BMBC, brain metastasis from breast cancer; BM, brain metastasis.

Among all patients, 45 (81.8%) had prescribed medication at time of diagnosis, while 33 (60%) patients were taking 3 or more daily medications.

### 3.3 Primary stage

Primary tumor stage was classified according to the TNM criteria, as far as feasible (30). Thirteen (27%), 18 (38%), 4 (9%), and 8 (17%) patients were diagnosed as having T1 (tumor size 1–20 mm), T2 (20–50 mm), T3 (>50 mm), and T4 tumors, respectively. The T stage for 5 (11%) patients could not be identified (Tx). The majority of patients (n = 33, 60%) had no metastasis at primary diagnosis (M0) and 17 (45%) had no locoregional lymph node involvement.

### 3.4 Histopathology

Among the abovementioned subtypes, the following were observed: luminal A-like (n = 1, 1.8%), luminal B-like (n = 20, 36.4%), TNBC (n = 11, 20%) and HER2-enriched (n = 8, 14.5%) subtypes. In 15 (27.3%) patients, the subtype could not be specified due to the lack of specific pathological information. Histopathological grading of the primary cancer revealed that 33 (60%), 12 (21.8%), and 2 (3.6%) patients had high-grade (G3) carcinoma, intermediate grade (G2) differentiation, and low-grade (G1) carcinoma, respectively.

### 3.5 Clinical presentation of brain metastasis at diagnosis

Forty-two (76%) patients had symptoms associated with brain metastasis at diagnosis. Six (11%) patients were asymptomatic at the time of diagnosis, and 7 (13%) patients had unclear symptoms at primary presentation.

### 3.6 Imaging

A midline shift was radiologically detected in 7 (13%) patients. In 6 (11%) patients, there were signs of intracranial pressure due to brain metastasis.

Radiographic brain metastasis distribution was complex, and no typical pattern was recognized. In most cases, metastases were localized in more than one anatomical brain region (n = 27, 49%). In 19 (34.5%), 5 (9%), and 1 (1.8%) patient, metastasis was localized exclusively in the cerebrum, the meninges, and the spine and cerebellum, respectively. In 2 patients (3.6%), the location of CNS metastases could not be retrospectively specified.

### 3.7 Treatment

Primary treatment of BMBC included radiotherapy only (e.g., whole-brain radiation (WBRT), stereotactic surgery (Stx-RT)) (38, 69%), surgery followed by radiotherapy (5, 9%), surgery alone (2, 4%), and chemotherapy (3, 5%). In 22 and eight patients WBRT and Stx-RT was applied. In eight patients, type of radiotherapy was not clear. There was no statistical difference in prognosis between WBRT vs. Stx-RT. (p=0.4545). One (2%) patient received hormonal therapy in combination with a CDK4/6 inhibitor. Six (11%) patients had no specific treatment assigned. After documented progression following initial treatment of brain metastasis, 11 (20%) patients received radiotherapy as a second treatment, 6 (11%) patients received systemic therapy, and 36 (65%) patients were assigned no treatment either because of their response to the initial treatment or due to the advanced state of disease without any benefit of further treatment. For one patient, further treatment was not documented.

### 3.8 Subgroup analysis

Our cohort was divided into 2 groups according to OS following BMBC diagnosis: OS <12 months and OS ≥12 months and subsequently labeled as short-term survivors (STS) and long-term survivors (LTS), respectively (**Table 2**). A total of 47 patients were identified in the 2 groups, among whom 29 (62%) survived less than 1 year and 18 (38%) more than 1 year from diagnosis of BMBC.

**Table 2:** Baseline characteristics of short-term and long-term survivors.

	OS <12 months	OS ≥12 months	*p*-value
All = 47	n = 29	n = 18	
Age at diagnosis of BC			
<65 years	21 (72.4%)	13 (72.2%)	
≥65 years	8 (27.6%)	5 (27.8%)	
Comorbidities			1.00
Yes	23 (79%)	14 (78%)	
No	6 (21%)	4 (22%)	
Medications			0.716
Yes	24 (83%)	14 (78%)	
No	5 (17%)	4 (22%)	
BC subtype			0.001
Luminal A	1 (3%)	0 (0%)	
Luminal B	11 (38%)	8 (44%)	
TNBC	10 (35%)	1 (6%)	
HER2/neu	0 (0%)	7 (39%)	
Unclear	7 (24%)	2 (11%)	
N-Stage at diagnosis of BC			0.141
N0	12 (50%)	2 (20%)	
N≥1	12 (50%)	8 (80%)	
M-Stage at diagnosis of BC			0.454
M0	19 (65%)	9 (50%)	
M1	10 (35%)	9 (50%)	
Pathological Grade			0.020
G1 (low grade)	1 (3.5%)	1 (6%)	
G2	4 (14%)	6 (33%)	
G3 (high grade)	23 (79%)	7 (39%)	
Unclear	1 (3.5%)	4 (22%)	
Anatomical site of BM			0.116
Cerebrum	12 (41.4%)	5 (27.8%)	
Cerebellum	0	1 (5.6%)	
Meningeal carcinomatosis	4 (13.8%)	0	
Spine	0	1 (5.6%)	
More than one site	12 (41.4%)	11 (61.1%)	
Unclear	1 (3.4%)	0	
Symptomatic BM			0.396
Yes	22 (75.9%)	14 (77.8%)	
No	5 (17.2%)	1 (5.6%)	
Unclear	2 (6.9%)	3 (16.7%)	
Signs of midline shift at diagnosis of BMBC			1.000
Yes	4 (14%)	3 (11%)	
No	25 (86%)	15 (89%)	
Signs of brain pressure at diagnosis of BMBC			1.000
Yes	4 (14%)	2 (11%)	
No	25 (86%)	16 (89%)	
Type of treatment of BMBC			0.239
Surgery	0	2 (11.1%)	
Radiotherapy	19 (65.5%)	12 (66.7%)	
Surgery followed by radiotherapy	2 (6.9%)	3 (16.7%)	
Chemotherapy	2 (6.9%)	0	
Unclear	1 (3.5%)	0	
No treatment	5 (17.2%)	1 (5.5%)	

**Table 2** shows a subgroup analysis of short-term survivors (OS <12 months after the diagnosis of BMBC) and long-term survivors (OS ≥12 months after the diagnosis of BMBC). BC, breast cancer; BMBC, brain metastasis from breast cancer; BM, brain metastasis; TNBC, triple-negative breast cancer.

In the LTS subgroup, the median age was 47 years (range 38–77). Fourteen (78%) patients had one comorbidity and were taking at least one prescription medication, while 9 (50%) were taking 3 or more medications at the time of diagnosis. In addition, only 2 (11%) patients had no locoregional lymph node involvement at the time of initial diagnosis, while 9 (50%) already had distant metastasis at the time of initial diagnosis.

Histopathologically, there were 8 (44%) luminal B-like BC cases, 7 (39%) HER2-positive cases, 1 (5.5%) TNBC case, and 2 (11%) unidentifiable molecular subtype cases in the LTS group. Seven (39%), 6 (33%), and 1 (5.5%) patient had a high-grade tumor (G3), moderately differentiated BC (G2), and low-grade tumor (G1), respectively. In 4 (22%) cases, grading could not be determined because of the lack of pathological data. Eleven (61%) patients had initial cerebral metastasis in more than one anatomic region; in 5 (28%) cases, metastasis was confined to the cerebrum, while in one (5.5%) case, metastases were found in the cerebellum and spine. In most cases in this subgroup, brain metastasis treatment included radiotherapy alone (12, 66%), surgical resection (2, 11%), resection followed by radiation (3, 17%), and no treatment (1, 5.5%).

The median survival in the STS group (OS <12 months) was 4.3 months, and that in the LTS group (OS ≥12 months) was 30.7 months (*p*<0.001). In the multivariate analysis, there were no statistically significant differences between the 2 groups in terms of comorbidities, medication use, M-stage, or symptomatology (**Table 2**). In addition, no significant differences adjusted for age (<65 years/≥65 years) or lymph node status (N0/N≥1) were detected.

We observed a significant difference (*p* = 0.001) in terms of the effect of molecular subgroup on OS. While a higher proportion (10, 34%) of TNBC was observed in the STS group than in the LTS group (1, 5.5%), HER2-positive subtypes were observed exclusively in the LTS group (7, 39%).

No significant difference (*p* = 0.02) was observed when adjusting for tumor grading. More G3 tumors were observed in the STS group (23, 79%) than in the LTS group (7, 39%).

Anatomical distribution and in particular disseminated cerebral metastasis compared to low-volume disease in the CNS had no statistically significant effect on survival (*p* = 1.0). Distribution according to therapy administration in the STS and LTS groups showed no notable differences with 65% and 66% respectively, having received radiotherapy alone. However, substantially more patients did not receive any therapy for BMBC in the STS group when compared with those in the LTS group (17% vs. 5.5%). Second-line therapy for BMBC showed significant (*p* = 0.003) differences in therapeutic modalities, with substantially more patients who received radiotherapy as a second-line therapy in the LTS group (N=8/44% vs. N=3/10.3%).

### 3.9 Survival analysis of risk groups

We compared the OS (defined as the time from diagnosis of BMBC to death or last follow-up) in 2 age groups (age <65 years (A) and ≥65 (B) years at diagnosis of BC). Further evaluations were made based on the presence or lack of symptoms of BMBC and disseminated and non-disseminated BMBC.

**Figure 1:**
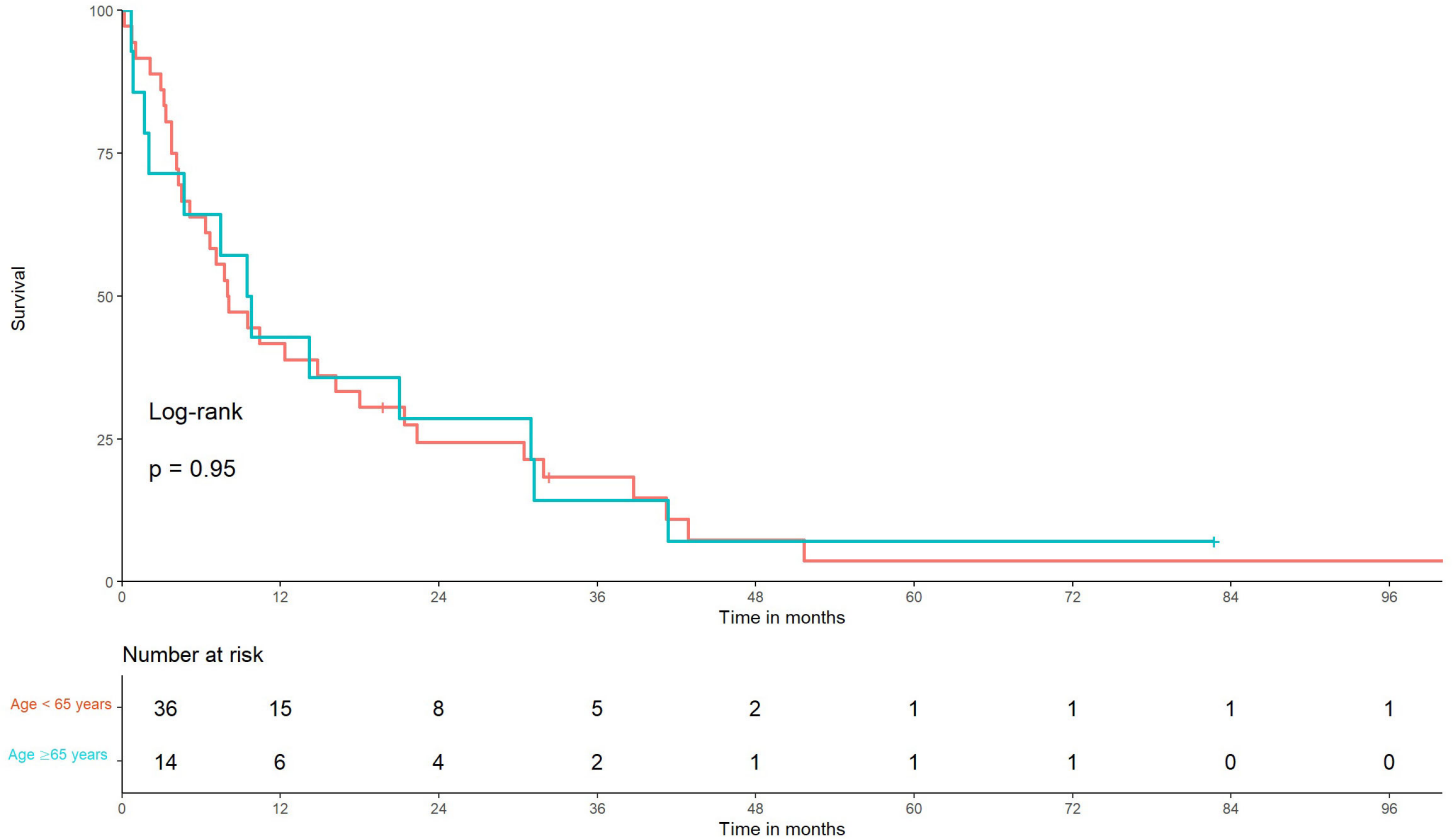
Kaplan–Meier graph of overall survival in older and younger patients with brain metastasis from breast cancer. Time from diagnosis of brain metastasis from breast cancer to death of patients younger than 65 years vs. those older than 65 years using Kaplan–Meier curves, *p* = 0.95.

**Figure 2:**
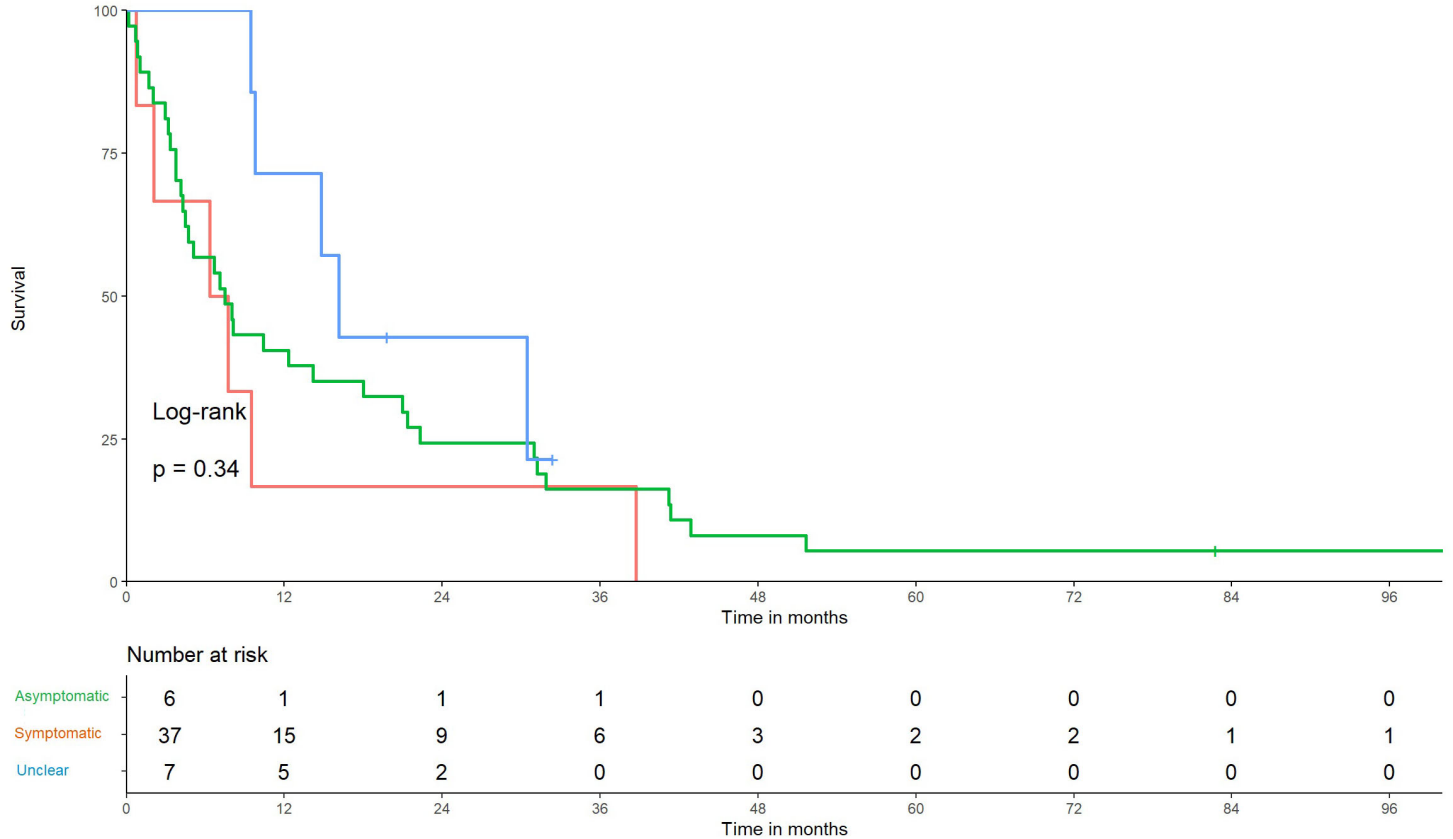
Kaplan–Meier graph of overall survival in patients with and without symptomatic brain metastasis. Survival of patients with a diagnosis of brain metastasis from breast cancer, defined as the time from diagnosis of brain metastases from breast cancer to death using Kaplan–Meier curves. No significant difference is noted between symptomatic and asymptomatic patients, *p* = 0.34.

Thirty-six patients in group A and 14 in group B were identified. The median OS was not significantly different (8.8 months in A, 95% CI: 5.1–18 vs. 9.6 months in B, 95% CI: 4.6–41) (*p* = 0.95), (**Figure 1**). With regard to symptoms of BMBC, 6 patients had asymptomatic disease, while 37 patients were symptomatic at the diagnosis of BMBC. No symptoms were identified in 7 patients. The median OS was 7.0, 7.4, and 16.1 months in the asymptomatic, symptomatic, and unclear BMBC manifestation groups, respectively. No statistically significant difference in OS was observed between patients with symptomatic and asymptomatic BMBC (*p* = 0.34) (**Figure 2**).

Moreover, we compared the median OS of patients with one or more brain metastasis to OS of those with radiologically identified multiple disseminated brain metastasis or meningeal carcinomatosis. Seventeen (35%) patients with disseminated and 32 (65%) patients with one or more brain metastases were identified. The median OS of patients with disseminated brain metastasis was 6.3 months, while that of patients with non-disseminated (≥1) BMBC was 9.4 months, with no statistical significance (*p* = 0.67) (**Figure 2**).

**Figure 3:**
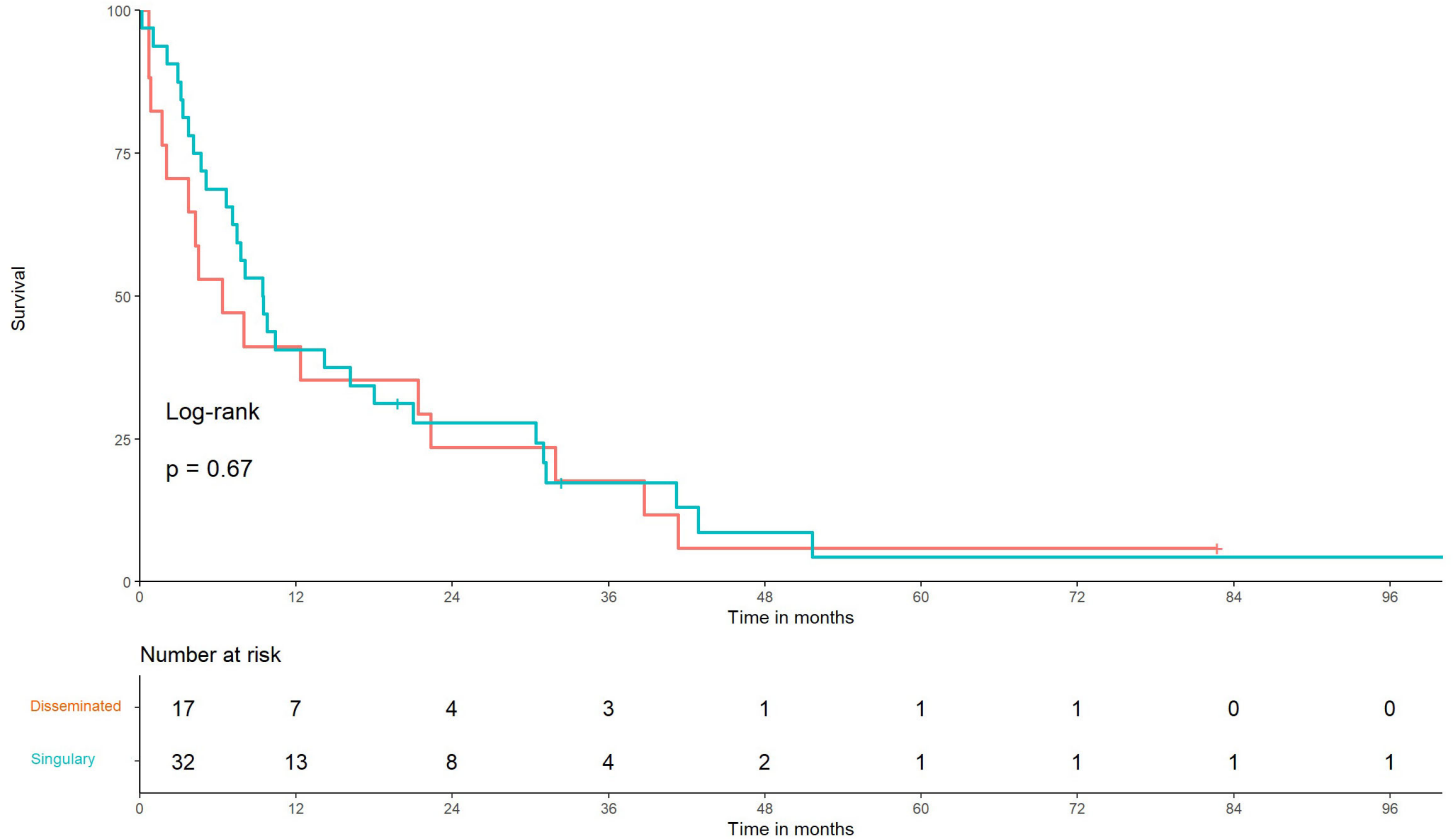
Kaplan–Meier graph of overall survival in patients with low- versus high-volume disease Time from diagnosis of brain metastasis from breast cancer (BMBC) to death of patients with multiple disseminated BMBC vs. single (≥1) BMBC using linear Cox regression, *p* = 0.67.

**Figure 4:**
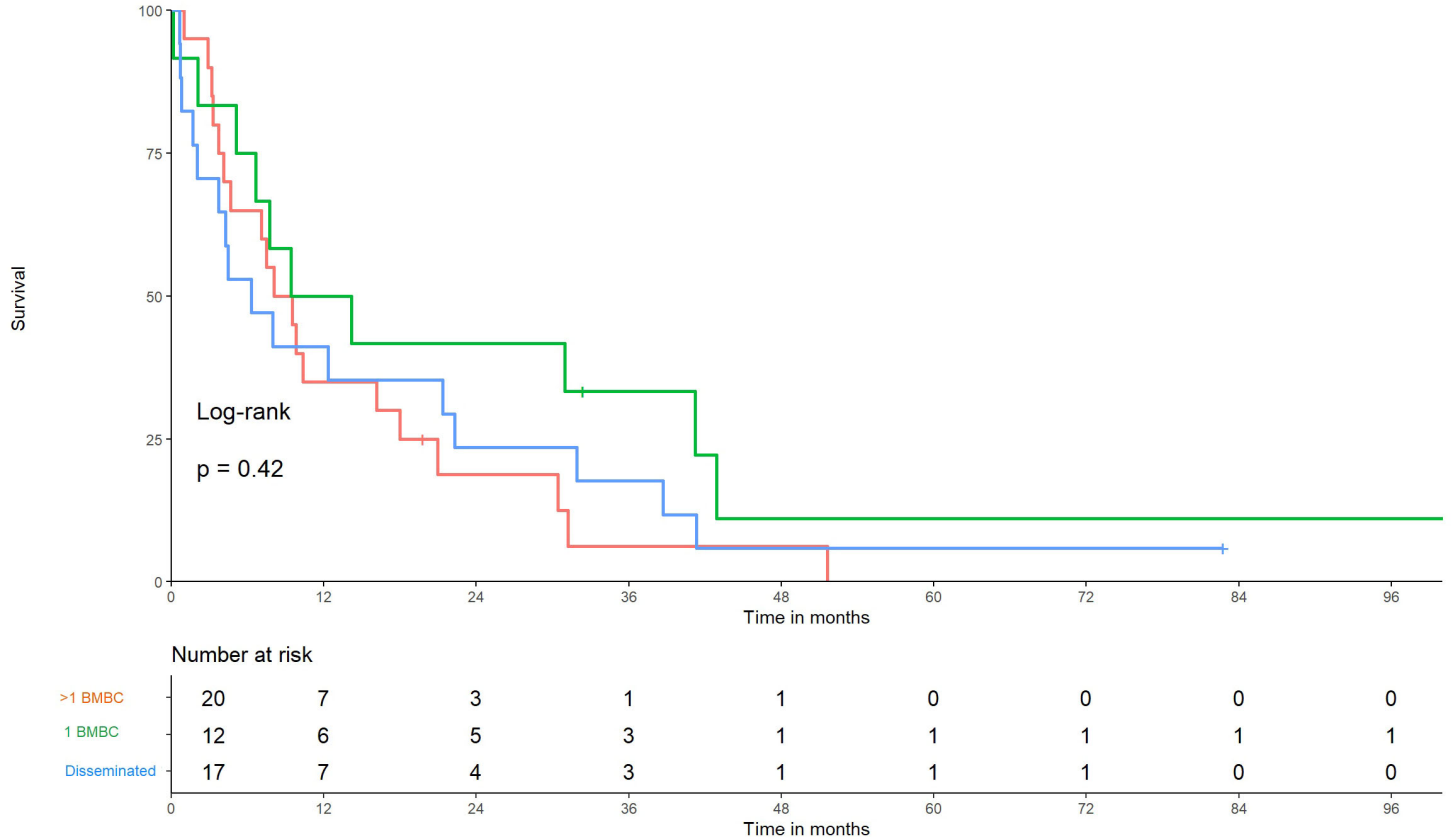
Kaplan–Meier graphs of overall survival in patients with single-site metastases versus low-volume versus high-volume disease. Time from diagnosis of brain metastases from breast cancer (BMBC) to death of patients with disseminated BMBC, 1 BMBC, and >1 BMBC, *p* = 0.42.

On comparing patients with one BMBC, >1 BMBC, and disseminated metastasis, we identified 20 (41%) patients with a single lesion, 12 (24%) with more than one, and 17 (35%) with disseminated brain metastasis (e.g., meningeal carcinomatosis). The median OS among these groups were 11.8, 8.8, and 6.2 months, respectively (*p* = 0.42) (**Figures 3**, **4**).

## 4 Discussion

There is a need for early detection and treatment for patients with BMBC. In this small retrospective chart review study, we identified specific factors that could be used to predict outcomes of patients with BMBC treated at our institution. According to the current literature, 5–50% of all patients with BC develop brain metastasis; however, only a small number of patients could be identified at our hospital (12–16). The relatively small number of patients diagnosed with BMBC could be explained by the relative abundance of treatment centers in a rather geographically confined region. As such, it is highly possible that after primary diagnosis, patients may have chosen different treatment sites based on their place of residence.

Overall, this study was illustrative for a heterogeneous group of patients, with most patients having HER2-positive and luminal B-like disease. Luminal A-like BC was significantly under-represented in our cohort compared with the proportion of all subtypes, as reported in the literature (only one patient in our study) (8, 31). According to the literature, luminal A-like tumors rarely develop metastasis and brain metastasis have a high cure rate (8, 26). However, interpretation of results is critical because much of the current decisive histological data at the time of diagnosis were not part of the generally accepted guidelines (32).

In our population, luminal B-like BC represented the largest group (36%), suggesting that elevated Ki-67 and/or HER2-positive status are risk factors for BMBC. These data are further supported by the findings of Heitz et al. and Sanna et al. who showed that HER2-positive patients have the highest risk of developing BMBC (10, 19). However, according to the literature, most BMBC cases are related to HER2 positive or TNBC subtypes not luminal BC (14, 25, 26). Whether this is due to the BC subtype not being available or recognized at the time of diagnosis or a lack of histological data (especially HER2 and Ki-67) in 15 (27%) patients, as described above, cannot be excluded with certainty.

Heitz et al. described primary non-metastatic disease as a risk factor for brain metastasis development (19). In our study, the majority (N=33, 60%) of patients had no distant metastasis (M0) at initial presentation. A possible explanation for this could be competing sites of metastasis such as in the lung or liver, contributing to overall shortening of OS before BMBC occurrence. Another mechanism by which late metastasis occurs, despite an initial M0 status, is a stock of dormant-disseminated or circulating cancer cells (CTCs), from which metastasis may develop later in the course of the disease. These were particularly found in the bone marrow of patients with BC (33–36). The prognostic relevance of CTCs in terms of recurrence and survival has already been demonstrated (36, 37). However, the relationship between the complex development of BMBC and the presence of CTC is not yet completely understood. Some genetic drivers of CTCs in the formation of brain metastasis in patients with BC have been documented; however, much research is needed for specific confirmation and routine test implementation (38).

Another clinical problem associated with the increased risk of developing BMBC is endocrine resistance in BC patients with ER+BC (39). 15-20% of ER+ BC are initially resistant while another 30-40% develop resistance to endocrine treatment over time (40). Lucke-Wold et al. showed in their review the need of specific systemic therapies in patients with endocrine resistance developing BMBC, especially monoclonal antibodies or CDK4/6 Inhibitors but the blood-brain barrier remains a major hurdle to the establishment of effective therapy for BMCB (41). In line with this, in our study we saw mostly local therapy (surgery, radiotherapy) options with chemotherapy being used in only 2 patients (once intrathecally).

In the hospital where the study was conducted, there are established treatment algorithms regarding BCBM. In particular, the BMBC patients are discussed in a tumor board to determine an optimal therapy following international guidelines. For singular BMBC, both neurosurgical resection and Stx-RT may be feasible. Stx-RT after resection can also be discussed, Mahajan et al. showed a lower local recurrence rate after stereotactic irradiation of completely resected brain metastasis in their study (42). WBRT was conducted in 22 patients while Stx-RT was done in only 8 patients. WBRT should be considered in patients with multiple BMBC not feasible for Stx-RT or Surgery due the possible effect on neurocognitive function (43). In our work 17 patients had disseminated BMBC which may be an explanation for the large proportion of WBRT in this study.

Systemic therapy for BCBM is also an important part of therapy. Especially in HER2-positive tumors, there are systemic therapy approaches for the treatment of BMBC: for example, the combination of capecitabine with lapatinib in the LANDSCAPE study showed objective response in BCBM (44). Furthermore, trastuzumab emtansine or pyrotinib have been shown to have encouraging results in the systemic treatment of BMBC of HER2-positive patients (45, 46). In our analysis, we could not detect any statistically significant prognostic factors for median OS. Analysis based on by age (cut-off 65 years) did not show any significant differences in terms of OS. In a previous study, a statistically relevant difference in OS was observed in patients aged <65 years in a similarly large population but only in patients whose BMBC was treated with a gamma knife (28). In contrast, this study included all treatment modalities (including none). Therefore, there are conflicting data regarding age as a possible prognostic factor (12, 47). Another study from Italy reported by Greto D et al. evaluates local treatment with gamma knife (48). In this study, patients with the age above 65 years had a worse outcome (HR 4.6). However, there was no clear difference in survival outcome for the number of treated brain metastasis. Overall, we think that the disease volume has an influence on prognosis but this is obviously depending on the included population of patients. Larger randomized studies comparing WBRT vs. Stx-RT are warranted.

The treatment of older patients with BMBC remains a challenge. Old age is associated with more comorbidities and frailty and does not reflect biological age. To optimize care for older patients with cancer, a systematic comprehensive geriatric screening assessment is warranted because it protects patients from under- and over-treatment. Geriatric screening was implemented in 2015 at our institution; however, regular geriatric comprehensive assessments are lacking, especially as this population has poor prognosis. Our data demonstrated the same outcome for patients older than 65 years, which could be biased as patients were selected based on their performance status. Unfortunately, we did not collect these data systematically.

Other selected variables also failed to show a relevant effect on OS. Interestingly, in our analysis, there were no differences in OS between patients with a single BMBC and those with disseminated cerebral metastasis (including meningeal carcinomatosis). The same percentage of patients (35%) in the LTS and STS groups showed disseminated metastasis regardless of OS. In a retrospective study, Thulin et al. showed a significantly longer OS in patients with 1–3 BMBC than in those with meningeal disease or with >3 BMBC, but no difference in OS was observed between patients with meningeal carcinomatosis and those with >3 BMBC (24).

The effect of disease volume on survival has also been shown through various studies (12, 28, 49). It is likely that this was not observed in our study because of the small number of patients. Regarding therapy of BMBC in the 2 groups (STS/LTS), the same proportion (66%) received radiotherapy but a higher proportion in the STS group received WBRT. However, more surgical resections (28% vs. 7%) were performed in in the LTS group. This indicates that patients in the STS group were in a generally poorer condition and/or had more advanced disease state because surgical resection is more invasive than radiotherapy alone. Unfortunately, the small number of patients does not allow a clear prognostic conclusion regarding OS and atamotic site of BMBC. In a literature review by Kancharla et al, however, no clear statement could be made regarding the relationship between anatomy and prognosis (50). As expected and described in literature, a high-grade (G3) tumor was correlated with shorter OS (12). As such, a higher proportion of G3 tumors (79%) was observed among patients in the STS group compared with that in the LTS group (39%). No statistically relevant prognostic factors were observed between the 2 subgroups. Performance status (e.g., Karnofsky performance status [KPS]) (12, 51) could not be accurately assessed owing to the lack of recorded data.

Unsurprisingly, a significantly larger proportion of patients with TNBC (10, 35%) were found in the STS group, while only one (6%) patient with TNBC lived longer than 12 months after the diagnosis of BMBC (*p* = 0.001). This was consistent with the overall poor prognosis of TNBC compared with those of other molecular BC subtypes (23, 52). For example, a retrospective single-center study showed a median OS of 4.9 months after the diagnosis of brain metastasis in patients with TNBC (16).

In contrast, patients with HER2-positive BC were observed only in the LTS group (N=7, 39%). In the literature, HER2-positive BC was associated with a high risk for the development of brain metastasis (10, 53). Conversely, according to the literature and consistent with our observations, better prognosis or OS was observed in patients with HER2-positive (HR-negative) BC and BMBC, respectively likely due to the above mentioned systemic treatment possibilities (43, 44, 47, 54)

Our study had limitations. The retrospective assessment of the study indicated that certain points could not be investigated, e.g., the KPS and G8 screening, and the small number of patients made statistical analysis difficult. This study is a retrospective cohort study, there was no overall-survival differences in patients that underwent WBRT or Stx-RT, but patients might be selected by performance status. Randomized data are needed to answer the question of best local treatment algorytms, especially also in older and frail patients.

However, this study aimed to illustrate the poor prognosis and unmet needs of patients with brain metastases and poor outcomes. Future trials and analyses should focus on the molecular basis of BC.

In conclusion, patients with BMBC had an overall poor prognosis; however, no statistically significant prognostic factors could be identified to suggest that BMBC is indicative of poor prognosis in populations with BC. In our study, age was deemed to be a relevant prognostic factor in treatment allocation for BMBC; however, because no effect was observed on general OS, our data suggest that all patients should be offered the same treatment modality, irrespective of age. It is important to determine the risk of brain metastasis and prevent its development. Based on our findings, BMBC is particularly important in patients who are positive for HER2 and who have TNBC and also in those with luminal B-like BC.

## Data availability statement

The raw data supporting the conclusions of this article will be made available by the authors, without undue reservation.

## Ethics statement

The studies involving humans were approved by EKNZ Ethikkomission Nord-West-Schweiz. The studies were conducted in accordance with the local legislation and institutional requirements. The ethics committee/institutional review board waived the requirement of written informed consent for participation from the participants or the participants’ legal guardians/next of kin (waived consent by the EKNZ Switzerland, EKNZ, Number 2020-03023).

## Author contributions

DD contributed to the conception, data collection, design of the article and drafted the first version of the manuscript. ED, MG, AZ, EC, CK and VD provided critical revisions of the manuscript. AS did the data analysis and provided critical revisions of the manuscript. MV contributed to conception and design of the article, provided critical revisions of the manuscript and supervision. All authors contributed to the article and approved the submitted version.

## References

[1] Statitik Bfür, BFS (2020). Available at: https://www.bfs.admin.ch/bfs/de/home/statistiken/gesundheit/gesundheitszustand/krankheiten/krebs/spezifische.html.

[2] Daily K Douglas E Romitti PA Thomas A. Epidemiology of *de novo* metastatic breast cancer. Clin Breast Cancer (2021) 21:302–8. doi: 10.1016/j.clbc.2021.01.017 33750642

[3] Malmgren JA Mayer M Atwood MK Kaplan HG. Differential presentation and survival of *de novo* and recurrent metastatic breast cancer over time: 1990–2010. Breast Cancer Res Treat (2018) 167:579–90. doi: 10.1007/s10549-017-4529-5 PMC5790843 29039120

[4] Disibio G French SW. Metastatic patterns of cancers: Results from a large autopsy study. Arch Pathol Lab Med (2008) 132:931–9. doi: 10.5858/2008-132-931-MPOCRF 18517275

[5] Pellegrino B Hlavata Z Migali C De Silva P Aiello M Willard-Gallo K. Luminal breast cancer: Risk of recurrence and tumor-associated immune suppression. Mol Diagn Ther (2021) 25:409–24. doi: 10.1007/s40291-021-00525-7 PMC8249273 33974235

[6] Pan H Gray R Braybrooke J Davies C Taylor C McGale P. 20-year risks of breast-cancer recurrence after stopping endocrine therapy at 5 years. N Engl J Med (2017) 377:1836–46. doi: 10.1056/NEJMoa1701830 PMC5734609 29117498

[7] Early Breast Cancer Trialists’ Collaborative Group. Aromatase inhibitors versus tamoxifen in early breast cancer: Patient-level meta-analysis of the randomised trials. Lancet (2015) 386:1341–52. doi: 10.1016/S0140-6736(15)61074-1 26211827

[8] Kennecke H Yerushalmi R Woods R Cheang MC Voduc D Speers CH. Metastatic behavior of breast cancer subtypes. J Clin Oncol (2010) 28:3271–7. doi: 10.1200/JCO.2009.25.9820 20498394

[9] Kumar P Aggarwal R. An overview of triple-negative breast cancer. Arch Gynecol Obstet (2016) 293:247–69. doi: 10.1007/s00404-015-3859-y 26341644

[10] Sanna G Franceschelli L Rotmensz N Botteri E Adamoli L Marenghi C. Brain metastases in patients with advanced breast cancer. Anticancer Res (2007) 27:2865–9. 17695462

[11] Weil RJ Palmieri DC Bronder JL Stark AM Steeg PS. Breast cancer metastasis to the central nervous system. Am J Pathol (2005) 167:913–20. doi: 10.1016/S0002-9440(10)61180-7 PMC1603675 16192626

[12] Rostami R Mittal S Rostami P Tavassoli F Jabbari B. Brain metastasis in breast cancer: A comprehensive literature review. J Neurooncol (2016) 127:407–14. doi: 10.1007/s11060-016-2075-3 26909695

[13] Barnholtz-Sloan JS Sloan AE Davis FG Vigneau FD Lai P Sawaya RE. Incidence proportions of brain metastases in patients diagnosed (1973 to 2001) in the Metropolitan Detroit Cancer Surveillance System. J Clin Oncol (2004) 22:2865–72. doi: 10.1200/JCO.2004.12.149 15254054

[14] Kuksis M Gao Y Tran W Hoey C Kiss A Komorowski AS. The incidence of brain metastases among patients with metastatic breast cancer: A systematic review and meta-analysis. Neuro-Oncology (2021) 23:894–904. doi: 10.1093/neuonc/noaa285 33367836 PMC8168821

[15] Lin NU Vanderplas A Hughes ME Theriault RL Edge SB Wong YN. Clinicopathologic features, patterns of recurrence, and survival among women with triple-negative breast cancer in the National Comprehensive Cancer Network. Cancer (2012) 118:5463–72. doi: 10.1002/cncr.27581 PMC3611659 22544643

[16] Lin NU Claus E Sohl J Razzak AR Arnaout A Winer EP. Sites of distant recurrence and clinical outcomes in patients with metastatic triple-negative breast cancer: High incidence of central nervous system metastases. Cancer (2008) 113:2638–45. doi: 10.1002/cncr.23930 PMC2835546 18833576

[17] Pestalozzi BC Holmes E de Azambuja E Metzger-Filho O Hogge L Scullion M. CNS relapses in patients with HER2-positive early breast cancer who have and have not received adjuvant trastuzumab: A retrospective substudy of the HERA trial (BIG 1-01). Lancet Oncol (2013) 14:244–8. doi: 10.1016/S1470-2045(13)70017-2 23414588

[18] Engel J Eckel R Aydemir U Aydemir S Kerr J Schlesinger-Raab A. Determinants and prognoses of locoregional and distant progression in breast cancer. Int J Radiat Oncol Biol Phys (2003) 55:1186–95. doi: 10.1016/s0360-3016(02)04476-0 12654426

[19] Heitz F Rochon J Harter P Lueck HJ Fisseler-Eckhoff A Barinoff J. Cerebral metastases in metastatic breast cancer: Disease-specific risk factors and survival. Ann Oncol (2011) 22:1571–81. doi: 10.1093/annonc/mdq625 21059640

[20] Voduc KD Cheang MC Tyldesley S Gelmon K Nielsen TO Kennecke H. Breast cancer subtypes and the risk of local and regional relapse. J Clin Oncol (2010) 28:1684–91. doi: 10.1200/JCO.2009.24.9284 20194857

[21] Lai R Dang CT Malkin MG Abrey LE. The risk of central nervous system metastases after trastuzumab therapy in patients with breast carcinoma. Cancer (2004) 101:810–6. doi: 10.1002/cncr.20418 15305414

[22] Niikura N Hayashi N Masuda N Takashima S Nakamura R Watanabe K. Treatment outcomes and prognostic factors for patients with brain metastases from breast cancer of each subtype: A multicenter retrospective analysis. Breast Cancer Res Treat (2014) 147:103–12. doi: 10.1007/s10549-014-3090-8 25106661

[23] Hines SL Vallow LA Tan WW McNeil RB Perez EA Jain A. Clinical outcomes after a diagnosis of brain metastases in patients with estrogen- and/or human epidermal growth factor receptor 2-positive versus triple-negative breast cancer. Ann Oncol (2008) 19:1561–5. doi: 10.1093/annonc/mdn283 18534964

[24] Thulin A Rönnerman E Zhang C De Lara S Chamalidou C Schoenfeldt A. Clinical outcome of patients with brain metastases from breast cancer – A population based study over 21 years. Breast (2020) 50:113–24. doi: 10.1016/j.breast.2020.02.007 PMC7375610 32145571

[25] Kim JS Kim K Jung W Shin KH Im SA Kim HJ. Survival outcomes of breast cancer patients with brain metastases: A multicenter retrospective study in Korea (KROG 16-12). Breast (2020) 49:41–7. doi: 10.1016/j.breast.2019.10.007 PMC7375558 31677532

[26] Nam BH Kim SY Han HS Kwon Y Lee KS Kim TH. Breast cancer subtypes and survival in patients with brain metastases. Breast Cancer Res (2008) 10:R20. doi: 10.1186/bcr1870 18307763 PMC2374976

[27] He DJ Yu DQ Wang QM Yu ZY Qi YH Shao QJ. Breast cancer subtypes and mortality of breast cancer patients with brain metastasis at diagnosis: A population-based study. Inquiry (2021) 58:469580211055636. doi: 10.1177/00469580211055636 34789038 PMC8619743

[28] Bir SC Bollam P Nanda A. Outcomes and predictors of improved survival after gamma knife radiosurgery for metastatic brain tumors originated from breast carcinoma. Neurosurg Rev (2015) 38:489–98. doi: 10.1007/s10143-015-0624-4 25843300

[29] Goldhirsch A Wood WC Coates AS Gelber RD Thürlimann B Senn HJ. Strategies for subtypes-dealing with the diversity of breast cancer: Highlights of the St. Gallen international expert consensus on the primary therapy of early breast cancer 2011. Ann Oncol (2011) 22:1736–47. doi: 10.1093/annonc/mdr304 PMC3144634 21709140

[30] Brierley J Gospodarowicz MK Wittekind C. TNM classification of malignant tumours. (2017) Wiley, Chichester.

[31] Jonnada PK Sushma C Karyampudi M Dharanikota A. Prevalence of molecular subtypes of breast cancer in India: A systematic review and meta-analysis. Indian J Surg Oncol (2021) 12 Supplement 1:152–63. doi: 10.1007/s13193-020-01253-w PMC8119535 33994741

[32] Li J Chen Z Su K Zeng J. Clinicopathological classification and traditional prognostic indicators of breast cancer. Int J Clin Exp Pathol (2015) 8:8500–5. PMC4555752 26339424

[33] Ren Q Khoo WH Corr AP Phan TG Croucher PI Stewart SA. Gene expression predicts dormant metastatic breast cancer cell phenotype. Breast Cancer Res (2022) 24:10. doi: 10.1186/s13058-022-01503-5 35093137 PMC8800302

[34] Sosa MS Bragado P Aguirre-Ghiso JA. Mechanisms of disseminated cancer cell dormancy: An awakening field. Nat Rev Cancer (2014) 14:611–22. doi: 10.1038/nrc3793 PMC4230700 25118602

[35] Harper KL Sosa MS Entenberg D Hosseini H Cheung JF Nobre R. Mechanism of early dissemination and metastasis in Her2+ mammary cancer. Nature (2016) 540:588–92. doi: 10.1038/nature20609 PMC5471138 27974798

[36] Braun S Vogl FD Naume B Janni W Osborne MP Coombes RC. A pooled analysis of bone marrow micrometastasis in breast cancer. N Engl J Med (2005) 353:793–802. doi: 10.1056/NEJMoa050434 16120859

[37] Bidard FC Vincent-Salomon A Gomme S Nos C de Rycke Y Thiery JP. Disseminated tumor cells of breast cancer patients: A strong prognostic factor for distant and local relapse. Clin Cancer Res (2008) 14:3306–11. doi: 10.1158/1078-0432.CCR-07-4749 18519757

[38] Klotz R Thomas A Teng T Han SM Iriondo O Li L. Circulating tumor cells exhibit metastatic tropism and reveal brain metastasis drivers. Cancer Discovery (2020) 10:86–103. doi: 10.1158/2159-8290.CD-19-0384 31601552 PMC6954305

[39] Cacho-Díaz B Spínola-Maroño H Arrieta VA Granados-García M Wegman-Ostrosky T Mendoza-Olivas LG. Diagnosis of brain metastases in breast cancer patients resulting from neurological symptoms. Clin Neurol Neurosurg (2018) 173:61–4. doi: 10.1016/j.clineuro.2018.08.002 30086430

[40] Lei JT Anurag M Haricharan S Gou X Ellis MJ. Endocrine therapy resistance: new insights. Breast (2019) 48 Suppl 1(Suppl 1):S26–30. doi: 10.1016/S0960-9776(19)31118-X PMC6939855 31839155

[41] Willman M Willman J Lucke-Wold B. Endocrine resistant breast cancer: brain metastasis. Explor Target Antitumor Ther (2022) 3(2):240–51. doi: 10.37349/etat.2022.00081 PMC9060566 35505937

[42] Palmer JD Klamer BG Ballman KV Brown PD Cerhan JH Anderson SK. Association of long-term outcomes with stereotactic radiosurgery vs whole-brain radiotherapy for resected brain metastasis: A secondary analysis of the N107C/CEC.3 (Alliance for clinical trials in oncology/Canadian cancer trials group) randomized clinical trial. JAMA Oncol (2022) 8(12):1809–15. doi: 10.1001/jamaoncol.2022.5049 PMC9585461 36264568

[43] Bachelot T Romieu G Campone M Diéras V Cropet C Dalenc F. Lapatinib plus capecitabine in patients with previously untreated brain metastases from HER2-positive metastatic breast cancer (LANDSCAPE): a single-group phase 2 study. Lancet Oncol (2013) 14(1):64–71. doi: 10.1016/S1470-2045(12)70432-1 23122784

[44] Mahajan A Ahmed S McAleer MF Weinberg JS Li J Brown P. Post-operative stereotactic radiosurgery versus observation for completely resected brain metastases: a single-centre, randomised, controlled, phase 3 trial. Lancet Oncol (2017) 18(8):1040–8. doi: 10.1016/S1470-2045(17)30414-X PMC5560102 28687375

[45] Jacot W Pons E Frenel JS Guiu S Levy C Heudel PE. Efficacy and safety of trastuzumab emtansine (T-DM1) in patients with HER2-positive breast cancer with brain metastases. Breast Cancer Res Treat (2016) 157:307–18. doi: 10.1007/s10549-016-3828-6 27167986

[46] Kioutchoukova I Lucke-Wold BP. Pyrotinib as a therapeutic for HER2-positive breast cancer. Transl Cancer Res (2023) 12(6):1376–9. doi: 10.21037/tcr-23-333 PMC10331451 37434682

[47] Miller JA Kotecha R Ahluwalia MS Mohammadi AM Chao ST Barnett GH. Overall survival and the response to radiotherapy among molecular subtypes of breast cancer brain metastases treated with targeted therapies. Cancer (2017) 123:2283–93. doi: 10.1002/cncr.30616 28192598

[48] Greto D Scoccianti S Compagnucci A Arilli C Casati M Francolini G. Gamma Knife Radiosurgery in the management of single and multiple brain metastases. Clin Neurol Neurosurg (2016) 141:43–7. doi: 10.1016/j.clineuro.2015.12.009 26731463

[49] Karakaya S Karadag I Ates O Cakmak Oksuzoglu OBC Demirci U. Clinical outcomes and prognostic factors in HER-2 positive breast cancer with brain metastasis: A single-centre experience. J Coll Physicians Surg Pak (2021) 31:166–70. doi: 10.29271/jcpsp.2021.02.166 33645183

[50] Kancharla P Ivanov A Chan S Ashamalla H Huang RY Yanagihara TK. The effect of brain metastasis location on clinical outcomes: A review of the literature. Neurooncol Adv (2019) 1(1):vdz017. doi: 10.1093/noajnl/vdz017 32642653 PMC7212918

[51] Sperduto PW Kased N Roberge D Xu Z Shanley R Luo X. Summary report on the graded prognostic assessment: An accurate and facile diagnosis-specific tool to estimate survival for patients with brain metastases. J Clin Oncol (2012) 30:419–25. doi: 10.1200/JCO.2011.38.0527 PMC3269967 22203767

[52] Sørlie T Perou CM Tibshirani R Aas T Geisler S Johnsen H. Gene expression patterns of breast carcinomas distinguish tumor subclasses with clinical implications. Proc Natl Acad Sci U.S.A. (2001) 98:10869–74. doi: 10.1073/pnas.191367098 PMC58566 11553815

[53] Darlix A Louvel G Fraisse J Jacot W Brain E Debled M. Impact of breast cancer molecular subtypes on the incidence, kinetics and prognosis of central nervous system metastases in a large multicentre real-life cohort. Br J Cancer (2019) 121:991–1000. doi: 10.1038/s41416-019-0619-y 31719684 PMC6964671

[54] Soffietti R Ahluwalia M Lin N Rudà R. Management of brain metastases according to molecular subtypes. Nat Rev Neurol (2020) 16:557–74. doi: 10.1038/s41582-020-0391-x 32873927

